# Neoadjuvant or Adjuvant Chemotherapy for Breast Cancer in Sub-Saharan Africa: A Retrospective Analysis of Recurrence and Survival in Women Treated for Breast Cancer at the Korle Bu Teaching Hospital in Ghana

**DOI:** 10.1200/GO.20.00664

**Published:** 2021-06-22

**Authors:** Hannah Ayettey Anie, Joel Yarney, Olutobi Sanuade, Shivanshu Awasthi, Tom Akuetteh Ndanu, Akash D. Parekh, Charles Aidoo, Mary Ann Dadzie, Verna Vanderpuye, Kosj Yamoah

**Affiliations:** ^1^ National Radiotherapy Oncology and Nuclear Medicine Centre, Korle Bu Teaching Hospital, Accra, Ghana; ^2^ Institute for Global Health, University College London, London, United Kingdom; ^3^ H. Lee Moffitt Cancer Center and Research Institute, University of South Florida, Tampa, FL; ^4^ University of Ghana Dental School, Accra, Ghana; ^5^ Department of Radiation Oncology, University of Florida, Gainesville, FL

## Abstract

It is established that addition of systemic therapy to locoregional treatment for breast cancer improves survival. However, reliable data are lacking about the outcomes of such treatment in women with breast cancer in low middle-income countries. We compared the outcomes of treatment in patients who had received neoadjuvant chemotherapy (NACT) or adjuvant chemotherapy and examined the factors associated with breast cancer recurrence and survival at the National Radiotherapy Oncology and Nuclear Medicine Centre, Korle Bu Teaching Hospital, Ghana.

**METHODS:**

This was a retrospective cohort study. The medical charts of women with breast cancer managed at the National Radiotherapy Oncology and Nuclear Medicine Centre from 2005 to 2014 were reviewed. A total of 388 patients with a median follow-up of 48 months were included in the study. Logistic regression was used to estimate the risk of recurrence. Survival was estimated using cox proportional hazards model. All models were adjusted with clinicopathologic variables. A *P* value of < .05 was considered statistically significant.

**RESULTS:**

Fifty-nine percent received adjuvant chemotherapy. In an adjusted logistic model, no difference was observed in locoregional recurrence between patients receiving NACT compared with those receiving adjuvant chemotherapy (odds ratio = 1.05; 95% CI, 0.44 to 2.47). However, NACT recipients had a higher likelihood of distant recurrence (odds ratio = 1.97; 95% CI, 1.24 to 3.15). In a multivariable analysis, no differences were observed in overall survival between the two chemotherapy groups (hazard ratio = 1.43; 95% CI, 0.91 to 2.26).

**CONCLUSION:**

NACT yields similar outcomes compared with adjuvant chemotherapy; however, recipients of NACT with advanced disease may have more distant failures. Early detection in a resource-limited setting is therefore crucial to optimal outcomes, significantly limiting recurrence and improving survival.

## INTRODUCTION

Breast cancer is the most common malignancy among women worldwide, representing 25% of all cancers in women and resulting in 14% of global deaths.^1^ In Ghana, breast cancer is the leading cause of cancer mortality among women. Although no official national registry exists in Ghana, it is estimated that in Korle Bu Teaching Hospital (KBTH) in Accra, Ghana, breast cancer accounts for 15.4% of all malignancies, and 33.9% in Kumasi.^2,3^ The Accra Cancer Registry, which is population-based, reports an age-standardized rate of 28.2 per 100,000 (unpublished literature). Despite campaigns to increase breast cancer awareness, data from Ghana and other sub-Saharan African countries indicate that women present late, often with more aggressive phenotypes and adverse features.^4-6^ Specifically, Ghanaian women typically have a higher proportion of triple-negative breast cancer (TNBC) compared with their White counterparts, more often present with stage III and IV disease, and are diagnosed at a younger age.^7^ Beyond normal barriers existing for all women undergoing breast cancer treatment, women in Ghana additionally face barriers to access, the lack of a national screening program, increased financial burden, social stigma, delay to treatment because of utilization of alternative treatment methodologies, and a lack of access to a cohesive medical network.^8^

**CONTEXT**

**Key Objective**
We compared the outcomes of treatment in patients with breast cancer who had received neoadjuvant or adjuvant chemotherapy and examined factors associated with recurrence and survival at the Korle Bu Teaching Hospital, Ghana. Despite international trials that confirm the equivalent survival of neoadjuvant chemotherapy (NACT) versus adjuvant chemotherapy, no current data exist comparing the general outcomes of breast cancer treatment in Ghana.
**Knowledge Generated**
NACT yielded similar outcomes compared with adjuvant chemotherapy; however, recipients of NACT with advanced disease had more distant failures. Although many women present with advanced-stage breast cancer in Ghana, outcome after trimodal therapy is reasonable for extent of disease.
**Relevance**
Despite significant resource constraints in Ghana, survival and recurrence rates for women with breast cancer treated with standard-of-care trimodal therapy are similar to those for patients in high-income countries. This study reinforces that early diagnosis and treatment of breast cancer is crucial to survival.

Given that more than 50% of women in Ghana present with advanced-stage disease, appropriate systemic therapy, which plays a key role in management of all stages of breast cancer, is a critical issue. In the absence of systemic treatment, the estimated 10-year risk of relapse approaches 35% in lymph node–negative disease.^9^ With systemic therapy, 10-year recurrence risk can be reduced to 20%.^9^

Neoadjuvant chemotherapy (NACT) and adjuvant chemotherapy provide equivalent survival benefit, but more women typically undergo adjuvant therapy across the globe.^10^ In contrast, in Ghana, there is anecdotal evidence that more women undergo NACT. NACT can help make inoperable disease operable and provide important information on the risk of future recurrence^10^; for example, women without a pathologic complete response to NACT have an increased risk of breast cancer mortality and distant recurrence (DR) than those with a complete response.^10^

Despite a varied number of international trials that confirm the equivalent survival of NACT versus adjuvant chemotherapy, no current data exist comparing the general outcomes of treatment in women with breast cancer in Ghana^11^ following NACT as compared with adjuvant chemotherapy. In addition, no study exists on factors associated with recurrence and survival. Without these data, it is impossible to compare outcomes with treatment elsewhere to determine the effectiveness of our treatment. Additionally, NACT is likely to be offered to more advanced disease relative to adjuvant treatment. To fill this critical gap, the present study examines the outcomes of treatment in patients who had received NACT and adjuvant chemotherapy and determines the factors associated with breast cancer recurrence and survival at the Korle Bu Teaching Hospital, Accra, one of the highest-volume tertiary care hospitals in sub-Saharan Africa.

## METHODS

This was a retrospective cohort study of female patients with breast cancer treated from 2005 to 2014 at the National Radiotherapy Oncology and Nuclear Medicine Centre, KBTH.

Patients with biopsy-proven breast cancer treated at the National Radiotherapy Oncology and Nuclear Medicine Centre between 2005 and 2014 were considered in the study. Of a total of 2,674 patients with breast cancer who were diagnosed during the study period, 388 met the inclusion criteria. Of those who did not meet the inclusion criteria, majority had metastatic disease. The inclusion criteria were as follows: (1) age 30-70 years, (2) T stages T1-T4, (3) all N stage, (4) invasive ductal or lobular carcinoma, and (5) must have undergone either NACT or adjuvant chemotherapy as part of their treatment. The following categories of patients were excluded from the study: (1) all male patients, (2) non-Ghanaians, (3) patients with metastatic disease, (4) patients with noninvasive cancer, (5) other breast cancer histopathologic subtypes, and (6) patients who did not complete their course of treatment, including chemotherapy and/or radiotherapy (RT).

The American Joint Committee on Cancer 8th edition was used to categorize patients into clinical and pathologic stage groups. Cancer groups IA-IIIA are categorized as early stage and IIIB and IIIC as advanced stage. Patients who were not in routine follow-up were contacted via telephone for updated disease status, and if deceased, to ascertain cause and date of death.

For this study, all patients who had started with at least one or more cycles of chemotherapy prior to surgery, and continued with adjuvant chemotherapy after surgery or completed NACT prior to surgery, were categorized as having had NACT. Patients who had chemotherapy solely after surgery were categorized as those who had adjuvant chemotherapy. Adjuvant chemotherapy usually consisted of six to eight cycles of chemotherapy dependent on the regimen given for NACT.

Breast-conserving therapy (breast conservation surgery plus RT) was offered to all patients who had wide local excision. Majority of patients who had radical modified mastectomy had indications for adjuvant RT and proceeded to have external beam RT. Indications for RT included T3/T4 disease, ≥ 4 positive nodes or > 20% positive nodes, positive deep resection margins, and following breast conservation surgery. The sequence of treatment was routinely NACT, followed by surgery and adjuvant chemotherapy (if not administered earlier) or following a few cycles (up to 2-4 cycles) of NACT and RT where appropriate. All patients with estrogen receptor–positive (ER+) and/or progesterone receptor–positive (PR+) disease received hormonal therapy, and human epidermal growth factor receptor 2 (HER2)–positive patients who could afford anti-HER2 therapy received trastuzumab for 9 weeks or 1 year depending on affordability, irrespective of the stage at presentation.

The disease-free survival (DFS), overall survival (OS), locoregional recurrence (LRR), and DR rates were calculated following data analysis. Clinicopathologic and sociodemographic factors associated with survival and recurrence rates were analyzed. LRR was measured as the date of first recurrent tumor of the same histology within the primary site or regional nodes in the absence of any prior recurrence. DR was defined as the diagnosis of DR in the absence of local or regional disease. OS was defined as the time from histopathologic diagnosis to death of any cause. DFS was defined as freedom from local recurrence, regional recurrence, or DR; cancer in contralateral breast; second primary; or death without evidence of recurrence. All end points are referenced from a start date of October 2016 as the baseline corresponding to the date of commencement of the study.

All data were analyzed using SAS 9.4 and SPSS version 22 (SPSS, Chicago, IL). Where applicable, appropriate descriptive statistics and frequencies were provided. Kaplan-Meier curves were developed to determine the survival measures, and the log-rank test was used to determine differences in survival. Cox proportional hazards model was used to perform univariate and multivariate analysis to determine factors associated with breast cancer OS and DFS. Logistic regression was performed for the variables that were either significant in the univariate model or not collinear with other predictors in the model to determine the odds of LRR and DR. The analysis plan was designed prior to data acquisition. The study was approved by the KBTH Institutional Review Board. The two-sided α value of < .05 was considered as statistically significant.

## RESULTS

### Patients' Characteristics

Demographic and pathologic tumor characteristics of the patient population including the NACT and adjuvant groups are displayed in Table 1. The median age of women included in the study was 51 years, with the largest proportion (35.1%) between 50 and 59 years. Most women were node-positive (73.4%). Approximately 29% of patients had triple-negative breast cancer. ER and PR were positive in 38.9% and 35.3%, respectively. HER2 was negative in more than half of the patients (55.9%), positive in about 17.5%, and equivocal in 4.4%. Surgical margins were negative for majority of the patients (80.4%). Lymphovascular invasion and perineural invasion were present in 20.6% and 4.6%, respectively.

**TABLE 1:**
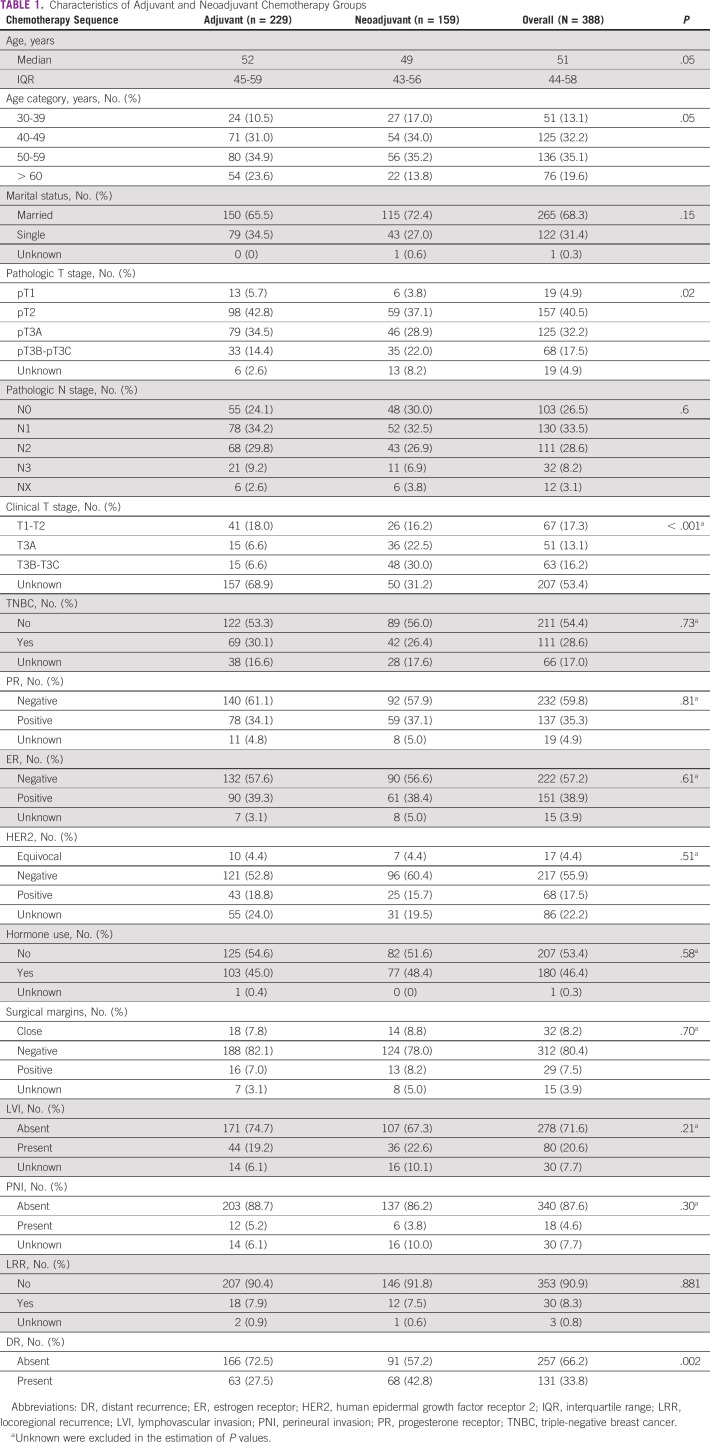
Characteristics of Adjuvant and Neoadjuvant Chemotherapy Groups

The two groups had comparable characteristics except that patients who submitted to NACT had larger tumors at presentation (*P* < .001) (Table 1). Fifty-nine percent (n = 229) received adjuvant chemotherapy, whereas 41.0% (n = 159) received NACT. Most women (94.1%, n = 365) had indications for adjuvant RT and received RT. Overall, 18.4% (n = 67) underwent breast conservation therapy followed by RT to a median dose of 50 Gy in 25 fractions, and 81.6% (n = 298) had a mastectomy followed by RT to a median dose of 50 Gy in 25 fractions. Of the patients who had NACT, 76 (47.8%) completed six cycles of chemotherapy, which included anthracycline-based regimens (cyclophosphamide, doxorubicin, and fluorouracil [FU]) (72.2%), anthracycline followed by taxanes (doxorubicin, cyclophosphamide, and paclitaxel) (23%), or antifolate agents (cyclophosphamide, methotrexate, and FU) (3.6%). Fifty-two percent (n = 83) did not complete six cycles of NACT, either at the preference of the treating physician, because of poor response to chemotherapy, or financial difficulties; hence, decision was made by the physician to proceed with surgery if tumor was resectable.

### OS and DFS Rates Following NACT or Adjuvant Chemotherapy

**FIG 1:**
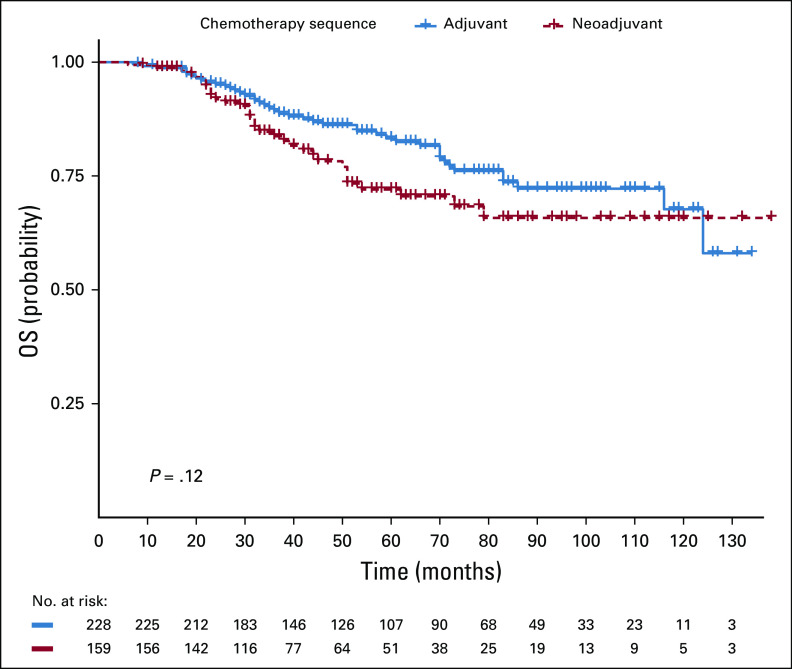
OS differences between adjuvant and neoadjuvant chemotherapy. OS, overall survival.

**FIG 2:**
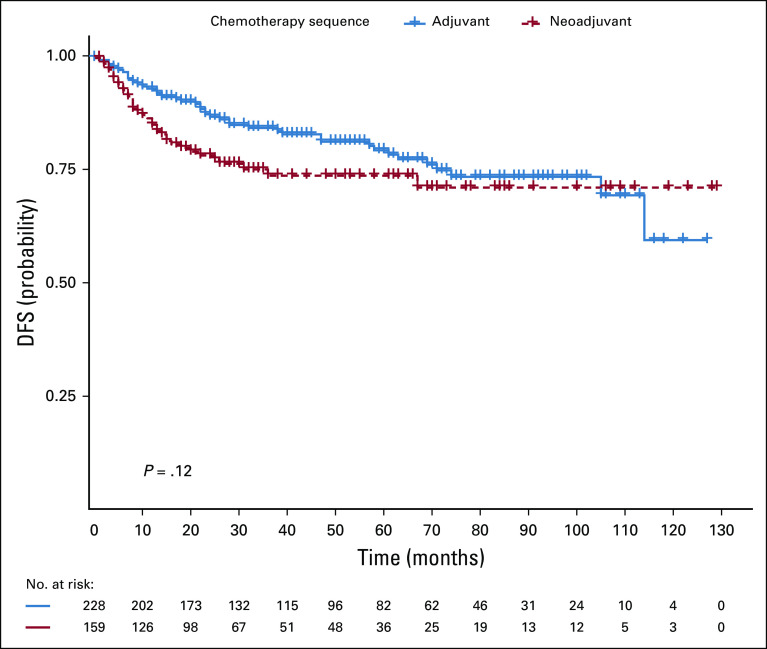
DFS differences between adjuvant and neoadjuvant chemotherapy. DFS, disease-free survival.

The median follow-up time was 48 months. The 2-, 5-, and 8-year OS rates were 95%, 91.5%, and 86.3% in the adjuvant group and 94.6%, 84.7%, and 77% in the NACT group, respectively (Fig 1). OS did not differ between the two groups (*P* > .05) (Table 3). The 2-, 5-, and 8-year DFS rates were 94.2%, 90.4%, and 85.2% for the adjuvant group and 95.1%, 80.1%, and 76.5% for the neoadjuvant group, respectively (Fig 2). This was not statistically different between the two groups (*P* = .1) (Table 3).

### LRR and DR Rates Following NACT or Adjuvant Chemotherapy

The cumulative proportion of LRR was 8.0% in the study population (Table 1). The locoregional rate was 7.9% in the adjuvant group compared with 7.5% in the neoadjuvant group (*P* > .05) (Table 1). The DR rate was significantly higher in the neoadjuvant group than in the adjuvant group (42.8% *v* 27.5%, respectively; *P* < 0.002) (Table 1).

### Clinical and Sociodemographic Predictors of Recurrence and Survival

In a univariate and multivariate analysis for LRR and DR, pathologic nodal (N) stage and clinical tumor (T) stage were associated with LRR. Increased DR was associated with NACT use, lack of hormone therapy use, higher clinical and pathologic T stage, node-positive disease, and lymphovascular invasion (Table 2). DR was significantly higher among the neoadjuvant group (42.8%) compared with the adjuvant chemotherapy group (27.5%) (Table 1). Inferior OS and DFS outcomes were associated with higher pathologic T and N stage, but not with estrogen- and progesterone-negative disease, and lack of hormone therapy use (Table 3). Worse DFS was additionally associated with the presence of lymphovascular invasion (Table 3). Pathologic T and N stage were statistically significantly associated with OS and DFS. On univariate analysis, no sociodemographic factors were associated with survival. Married women tended to have increased locoregional recurrence and DFS. About 19% of married women presented with locally advanced disease compared with 14.6% of single and divorced women. There was however no statistically significant correlation between these two groups.

**TABLE 2:**
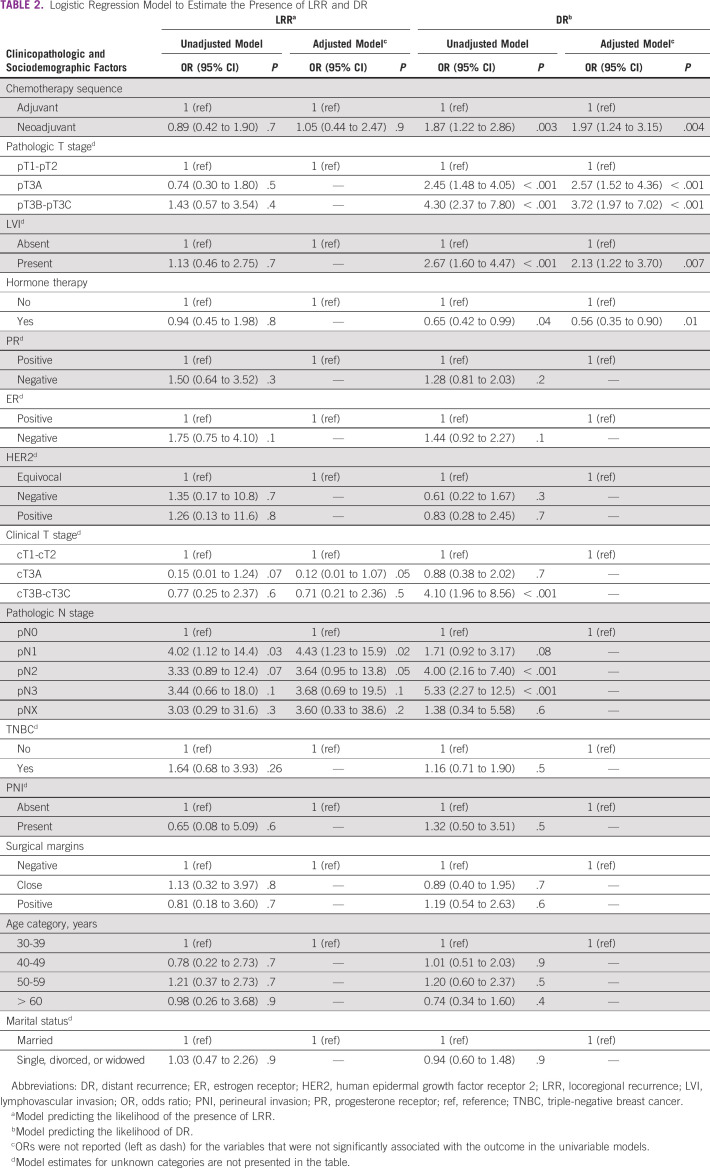
Logistic Regression Model to Estimate the Presence of LRR and DR

**TABLE 3:**
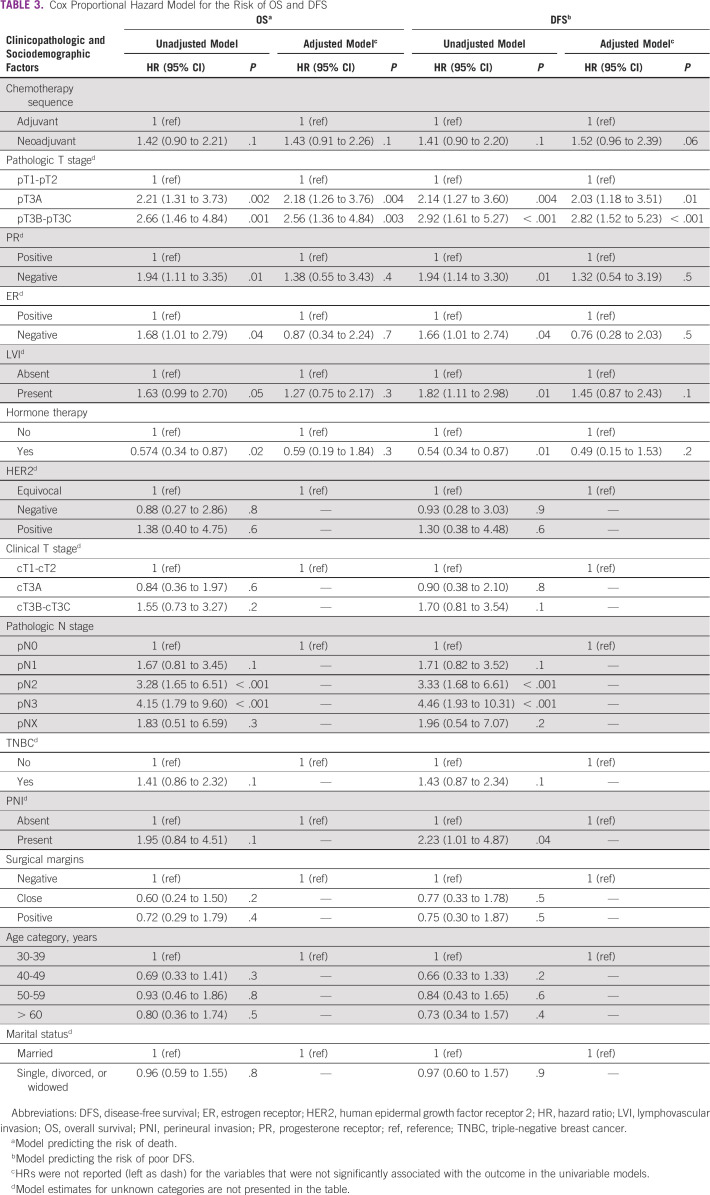
Cox Proportional Hazard Model for the Risk of OS and DFS

## DISCUSSION

This retrospective study set out to compare the outcomes of treatment in patients who had received NACT or adjuvant chemotherapy and examine the factors (clinicopathologic and sociodemographic) associated with breast cancer recurrence and survival at the Korle Bu Teaching Hospital, Accra, Ghana. In our study, we found that NACT was associated with a higher frequency of DR compared with chemotherapy administered after surgery, with a 10-year DR rate of 27.5% in the adjuvant group and 42.8% in the NACT group. Increase in DR did not translate into a survival disadvantage, probably because of second-line chemotherapy offered to these patients after documentation of recurrence. The LRR rate was similar between both groups, as was the OS and DFS. It is possible that a delay in definitive locoregional treatment administered to the NACT group or the variable number of chemotherapy cycles administered was a contributing factor to the increase in DR.

Additionally, the NACT group was found to have a greater proportion of late-stage patients, which could have translated into increased DR in that group. Patients with locally advanced disease are usually predetermined to undergo NACT to downstage the disease prior to surgery by the treating physician. A study particularly showed that circulating tumor cells in the peripheral blood of patients with large operable or locally advanced breast cancer was an independent prognostic factor for early relapse after NACT and this could be a contributory reason for high DR in this group.^12^ Another study looked at the profile of residual breast cancer after NACT and identified dual specificity protein phosphatase 4 deficiency as a mechanism for drug resistance and a consequent increased incidence of residual disease culminating in a higher risk of metastatic disease.^13^ This may also have contributed to the statistically significant association between NACT and increased DR rate, although this cannot be confirmed in the present study.

Many of the patients in our series who were older were either ER+ or PR+ and did not have TNBC. It is well-known that younger patients who have TNBC benefit most from NACT, whereas ER+ or PR+ patients derive the least benefit.

Results from the present study demonstrate OS, DFS, and recurrence rates comparable with larger studies in developed countries.^14^ Moreover, OS and DFS were similar between the NACT and adjuvant chemotherapy groups, which is consistent with the recently published Early Breast Cancer Trialists Collaborative Group (EBCTCG) meta-analysis.^15^ The authors additionally found that women undergoing NACT experienced higher LRR after breast-conserving therapy, but DR was similar between the two arms. As in the present study, survival was not adversely affected by higher recurrence. Furthermore, women whose tumors responded to NACT had improved breast cancer mortality and lower DRs than nonresponders.^15^ This suggests that appropriate tumor bed localization and RT following NACT will reduce the likelihood of LRR.

The results of the EBCTCG meta-analysis are similar to those of National Surgical Adjuvant Breast and Bowel Project-18, which looked at the impact of preoperative versus postoperative doxorubicin and cyclophosphamide on survival of women with operable breast cancers.^16^ The authors found no difference in DFS and OS between the arms. Eight-year OS and DFS were 72% and 55% in the adjuvant arm and 72% and 58% in the NACT arm, respectively. These values are comparable with the DFS and OS estimates in our cohort of patients. This is encouraging given the resource limitations present in our setting. There was a trend in favor of preoperative chemotherapy for OS and DFS in women younger than 50 years.^16^ The authors suggest that women < 50 years may benefit from preoperative chemotherapy, whereas those > 50 years may benefit from postoperative chemotherapy. This may explain why a DR benefit was observed in patients older than 50 years in our cohort. The results of National Surgical Adjuvant Breast and Bowel Project-18 are similar to those of European Organization for Research and Treatment of Cancer 10902.^16^ After randomly assigning women with operable breast cancer to either pre- or postoperative FU, epirubicin, and cyclophosphamide, there was no difference in OS, DFS, or LRR, and NACT resulted in a higher rate of breast conservation therapy without increasing LRR or decreasing survival. The lack of impact on locoregional control contrasted to the EBCTCG meta-analysis but is similar to our present findings.

Consistent with our finding that higher clinical T stage and N stage are associated with worse locoregional control, Mamounas et al^17^ found that age, clinical T stage and nodal involvement before chemotherapy, and tumor response were associated with LRR after NACT. In their study, only patients who underwent lumpectomy received breast RT. No postmastectomy irradiation was administered. The cohort of patients in that study had primary operable breast cancer. Although not directly comparable with our cohort, their findings enforce the importance of continued early detection of breast cancer.

One of our study objectives was to determine if any sociodemographic factors were associated with recurrence and survival. No sociodemographic factors were associated with survival. However, a higher percentage of married women recurred locoregionally and distantly. To our knowledge, only one other study has also examined the impact of marital status on survival.^18^ The authors found that married women were less likely to present with metastatic disease. Although this association could be because of multiple factors, these findings point to the importance of accounting for socioeconomic and familial challenges that may affect cancer detection, treatment, and survival in Ghana.^18^

Beyond obvious limitations inherent to all retrospective studies, most often related to incomplete documentation and poor follow-up, our study has other limitations. Information regarding clinical response to chemotherapy, hormone receptor status, and tumor grade was either incomplete or not possible to obtain. Unknown responses under histopathologic factors were excluded from the analysis. These may have biased the results. Reasons for prescribing NACT were sometimes not related to clinical factors but rather because of resource constraints and efforts to reduce waiting time prior to surgery. Despite these limitations, our study provides clinically relevant results on outcomes of treatment and factors associated with breast cancer recurrence and survival among Ghanaian women following NACT and adjuvant chemotherapy.

In conclusion, despite significant resource constraints in Ghana, survival and recurrence rates for women with breast cancer treated with standard-of-care trimodal therapy in the country are similar to those for patients in high-income countries. However, our data indicate that women undergoing NACT experienced a higher rate of DR compared with those undergoing adjuvant chemotherapy probably from more advanced disease at presentation. This did not translate into a survival disadvantage. No sociodemographic factors considered were associated with recurrence or survival. As is consistent with published studies, large tumor size and nodal involvement adversely affected survival and recurrence. This study reinforces that early diagnosis and treatment of women with breast cancer is crucial to survival. Continued effort should be made to reduce the culture of late presentation and treatment defaults in Ghana.
